# Effectiveness of joint 3 + 1 malaria strategy along China–Myanmar cross border areas

**DOI:** 10.1186/s12879-021-06920-z

**Published:** 2021-12-14

**Authors:** Zu-rui Lin, Shi-gang Li, Xiao-dong Sun, Xiang-rui Guo, Zhi Zheng, Jie Yang, Hong-ru Pian, Peng Tian, Qi-yan Chen, Xiao-ying Sun, Chun-li Ding, Kai-xia Duan, Hong-wei Chen, Dakhidam Yaw Bee, Hong-ning Zhou

**Affiliations:** 1 Yunnan Institute of Parasitic Diseases; Yunnan Provincial Centre of Malaria Research, Yunnan Provincial Collaborative Innovation Centre for Public Health and Disease Prevention and Control, Yunnan Provincial Key Laboratory of Vector-Borne Diseases Control and Research, Pu’er, 665000 China; 2 Yangjiang Centre for Disease Control and Prevention, Yangjiang, 679300 China; 3 Institute of Basic Medical Sciences, Chinese Academy of Medical Sciences, School of Basic Medicine, Peking Union Medical College, Beijing, 100730 China; 4 Dehong Centre for Disease Control and Prevention, Mangshi, 678400 China; 5 Health Poverty Action, Kunming, 650051 China; 6 Nangbang Township Central Hospital, Yingjiang, 679300 China; 7 Malaria Project Office, Health Department of Kachin Special Region II, Laiza City, Myanmar

**Keywords:** Malaria, China–Myanmar border, Cross, Joint, Strategy, Control, Elimination

## Abstract

**Background:**

Cross-border malaria in Laiza City of Myanmar seriously affected Yingjiang County of China and compromised reaching the goal of malaria elimination by 2020. Since 2017, a pilot project on 3 + 1 strategy of joint cross-border malaria prevention and control was carried out for building a malaria buffer in these border areas. Here, 3 were the three preventive lines in China where different focalized approaches of malaria elimination were applied and + 1 was a defined border area in Myanmar where the integrated measures of malaria control were adopted.

**Methods:**

A 5-year retrospective analysis (2015 to 2019) was conducted that included case detection, parasite prevalence and vector surveillance. Descriptive statistics was used and the incidence or rates were compared. The annual parasite incidence and the parasite prevalence rate in + 1 area of Myanmar, the annual importation rate in Yingjiang County of China and the density of *An. minimus* were statistically significant indictors to assess the effectiveness of the 3 + 1 strategy.

**Results:**

In + 1 area of Myanmar from 2015 to 2019, the averaged annual parasite incidence was (59.11 ± 40.73)/1000 and *Plasmodium vivax* accounted for 96.27% of the total confirmed cases. After the pilot project, the annual parasite incidence dropped 89% from 104.77/1000 in 2016 to 12.18/1000 in 2019, the microscopic parasite prevalence rate dropped 100% from 0.34% in 2017 to zero in 2019 and the averaged density of *An. Minimus* per trap-night dropped 93% from 1.92 in June to 0.13 in September. The submicroscopic parasite prevalence rate increased from 1.15% in 2017 to 1.66% in 2019 without significant difference between the two surveys (*P* = 0.084). In Yingjiang County of China, neither indigenous nor introduced case was reported and 100% cases were imported from Myanmar since 2017. The averaged annual importation rate from 2015 to 2019 was (0.47 ± 0.15)/1000. After the pilot project, the annual importation rate dropped from 0.59/1000 in 2016 to 0.28/1000 in 2019 with an overall reduction of 53% in the whole county. The reduction was 67% (57.63/1000 to 18.01/1000) in the first preventive line, 52% (0.20/1000 to 0.10/1000) in the second preventive line and 36% (0.32/1000 to 0.22/1000) in the third preventive line. The averaged density of *An. Minimus* per trap-night in the first preventive line dropped 94% from 2.55 in June to 0.14 in September, without significant difference from that of + 1 area of Myanmar (Z value = − 1.18, *P* value = 0.24).

**Conclusion:**

The pilot project on 3 + 1 strategy has been significantly effective in the study areas and a buffer zone of border malaria was successfully established between Laiza City of Myanmar and Yingjiang County of China.

**Supplementary Information:**

The online version contains supplementary material available at 10.1186/s12879-021-06920-z.

## Background

Although, significant progress has been documented in the National Malaria Elimination Programme, cross-border/imported malaria remains a major challenge in malaria elimination in China. China 1–3–7 malaria strategy which refers case reporting within 1 day, case confirmation and investigation within 3 days, and focus response within 7 days has led to significant achievements and milestones in malaria control to elimination [1–3]. Its performance and key technical specification had remarkable impact in shrinking the national map of indigenous malaria from 762 to 2 counties and with 4262 cases to 3 cases from 2010 to 2016 respectively [4, 5]. In 2016, the World Health Organization (WHO) identified 21 countries, including China, as those on track to reach the goal of malaria elimination by 2020 [6].

Yunnan Province of China (YNC) shares a land border of 4060 km with Myanmar, Lao PDR and Vietnam in the Greater Mekong Sub-region. Among them, 18 counties in the West of YNC are adjacent to 5 special regions in the North of Myanmar with a land border of 1997 km. Myanmar is still one of the counties with highest malaria burden in the world [6], especially in the 5 special northern regions due to the limited health resources [7, 8]. In the 18 counties of YNC, most malaria cases are imported from the neighbor owing to intensive and regular daily cross-border activities. Consequently, curbing and controlling China–Myanmar cross-border malaria has become a major challenge to malaria elimination in YNC [9–11].

WHO defines border malaria as malaria transmission or potential transmission that takes place across or along boundaries between countries sharing a land border, it includes movement of infected people crosses boundaries, and/or mosquito transmission crosses or occurs along boundaries [12]. In order to reduce the malaria burden in China–Myanmar border area, an effective joint cross-border malaria prevention and control mechanism was set up between YNC and Myanmar since 2007, supported by the sixth and tenth round of China malarial project in the Global Fund to Fight AIDS, Tuberculosis and Malaria (refer to as GF malaria program) [13–20]. It was comprised of bilateral malaria programs and capacity transfer, as well as exchange of epidemic information [13, 15, 17], YNC’s provision of malaria capacity transfer and training, and establishment of 66 malaria consultation posts in the 18 border counties to detect and treat malaria for entry people, and to deliver a malaria protective pack for exit people [19, 20]. Myanmar’s establishment of 80 malaria medical stations in the 5 special regions was to carry out malaria case detection and management, provide distribution of long-lasting insecticidal nets (LLINs) or insecticide treated nets (ITNs) and promote health education [14, 16]. These integrated approaches led to significant achievements including the reduction of annual parasite incidence (API) from 41.7/1000 in 2008 to 7.1/1000 in 2013 in Myanmar side, and from 1.9/1000 in 2006 to 0.09/1000 in 2013 in China side. The reduction in malaria burden was 89% and 95%, respectively [20]. However, these milestones and achievements were not sustained due to the GF Malaria Program interruption for China in January 2014. As a remedy a high-level bilateral memorandum of understanding between China and Myanmar was signed in Tengchong City of YNC in 2016 and a new joint action plan for fighting malaria in both border areas was proposed to accelerate malaria elimination in China and rollback malaria in Myanmar.

Laiza City (LZC) of Myanmar is the political center of Kachin Special Region II (KSR II) and shares a land border of 20.5 km with Nabang Township of Yingjiang County (YJC) of YNC. After the regional conflict in KSR II in 2012, more than 30,000 internally displaced persons (IDP) migrated to LZC and resettled along the boundary. This greatly aggravated the malaria burden in both sides, resulting in the malaria incidence of KSR II rebounding from 2.1% in 2012 to 5.1% in 2016 [18], and the number of reported malaria cases in YJC sharply raised from 58 (23 indigenous cases) in 2012 [15] to 186 (1 indigenous case) in 2016, which accounted for 59.23% of the total reported cases in YNC in 2016. This was the only county where the number of reported cases raised rather than dropped since the National Malaria Elimination Programme launched in YNC in 2010 and local transmission continued to 2016 [5]. YJC has become the most challenging areas for achieving the goal of malaria elimination in China.

A pilot new joint cross-border malaria prevention and control project termed 3 + 1 strategy was initiated for establishing a buffer zone of border malaria in these areas since 2017. Here, + 1 was a catchment area in LZC of Myanmar (refer to as + 1 area), a defined region with a length of 20.5 km and a width of 2.5 km along the boundary, where the implementation of joint working group and adoption of integrated malaria control and elimination measures were aimed at reinforcing the existing local GF malaria programs. That included funding and technical assistance, capacity transfer and training early case detection and management, focus response, vector surveillance and control, health education, and radical treatment. 3 were to establish three preventive lines (TPLs) in YJC of China and to take the focalized approaches of national 1–3–7 strategy in each line. The first preventive line (1st PL) was the same defined region in the border areas of YJC corresponding to + 1 area of Myanmar, where the core interventions were vector surveillance and control, entry-exit person management, reactive and proactive case detection aiming at reducing the risk of case importation and local transmission interruption. The second preventive line (2nd PL) included other border areas in YJC besides 1st PL with core interventions including proactive malaria case detection and mobile population management in preventing transmission re-establishment. The third preventive line (3rd PL) covered the non-border areas including downtown of YJC where the core interventions were passive case detection for early detection and prompt treatment to maintain zero indigenous case.

This study was to assess the impact of joint 3 + 1 strategy and to share the learned experiences and lessons for accelerating malaria elimination agenda in the Greater Mekong Sub-region.

## Methods

### Study areas and population

**Fig. 1:**
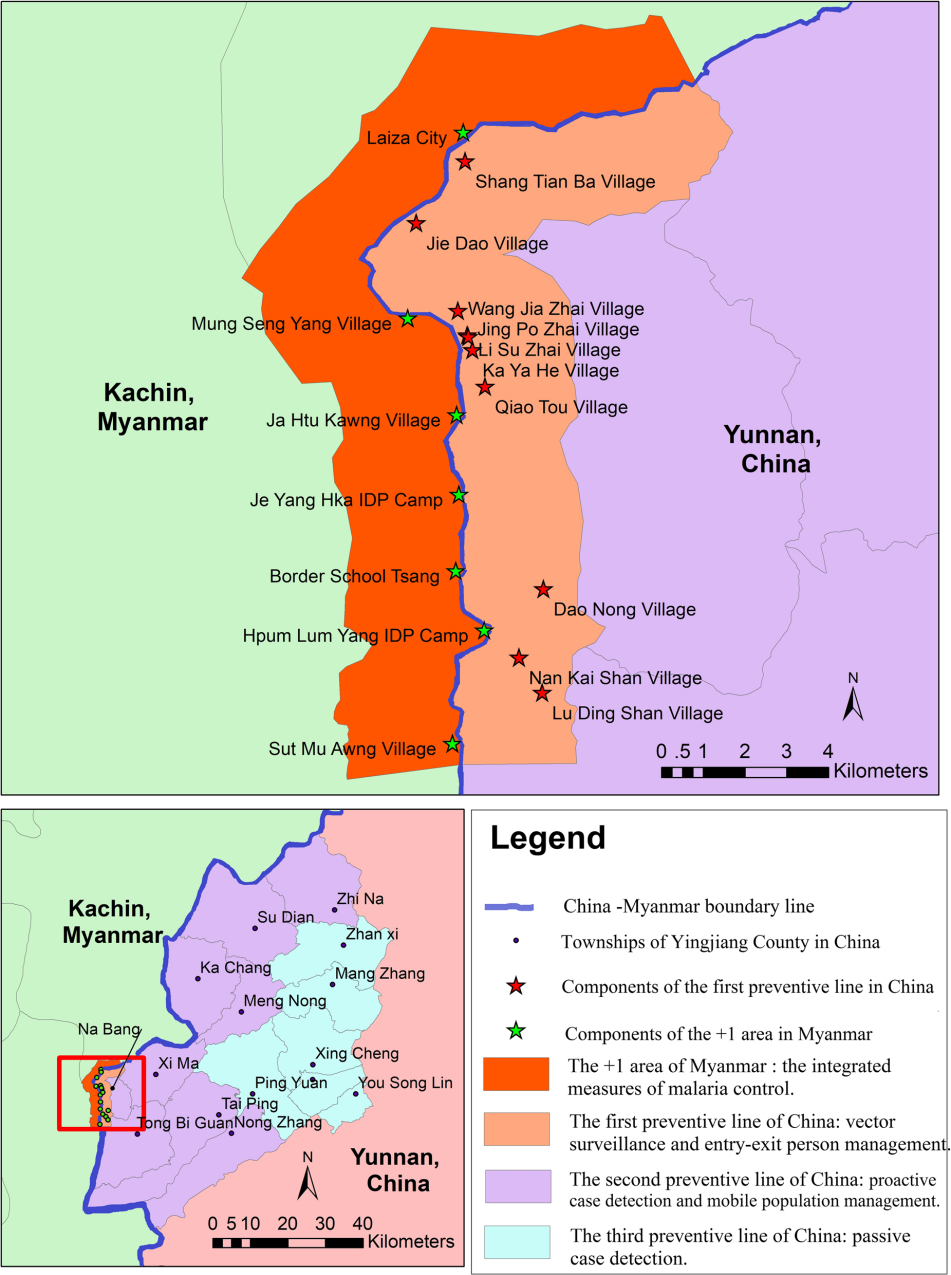
The map of study area of Pilot project on 3 + 1 malaria strategy. The map was depicted by us with ArcMap 10.7 version

The township and its county where the most imported malaria cases were reported in YNC in 2016, as well as the region of Myanmar within 2.5 km deep from the border line with the enrolled township of YNC were selected as the study areas. The study areas were the catchment areas of pilot project based on 3 + 1 strategy. 1st PL of YJC covered all 10 natural villages in Nabang Townships with population of about 2000. 2nd PL covered 51 administrative villages in 8 border townships of YJC with population about 147,000. 3rd PL covered 27 administrative villages in 6 non-border townships and downtown of YJC with population about 160,000. + 1 area of Myanmar covered the urban city of LZC, 4 natural villages, a high school and 2 camps of IDP, with a population about 20,000. Figure 1 shows a map of the study area of the pilot project on 3 + 1 strategy.

### Data resources and collection

#### Case detection

In LZC of Myanmar, the data of routine malaria and population size from January 1st, 2015 to December 31st, 2019 were collected from the office of GF malaria program in KSR II that monthly collected at grass-root health facilities via the health information system which established and maintained by GF malaria program since 2007 [20]. Data of confirmed cases and blood examination in + 1 area were extracted from the monthly report of Laiza central hospital (that including the data of LZC hospital), the camp clinic of Je Yang Hka and the camp clinic of Hpum Lum Yang. In YJC of China, Data of registered malaria cases from January 1st, 2015 to December 31st, 2019 were collected from the information management system specific to malaria elimination [1] and nationwide notifiable infectious diseases reporting information system [21]. The data of blood examination were extracted from the monthly report of each sentinel hospital that collected by County Center for Disease Control and Prevention (CDC) as part of GF malaria program management since 2003. Data of population size were extracted from the statistical yearbook of YJC (Additional file 1).

#### Determination of parasite prevalence

The parasite prevalence rate of + 1 area of Myanmar was surveyed at the beginning (April 2017) and the end (December 2019) of the pilot project in the 2 IDP camps, using a novel real-time quantitative PCR-based technique termed capture and ligation probe-PCR (CLIP-PCR) [22, 23]. Each participant’s body temperature was measured and 2 dried blood spots were collected. If the body temperature was higher than 37.5 °C or a fever occurred within the past week, the participant was immediately tested with a rapid diagnosis test (RDT, Wondfo Pf/Pan-LDH, China, lot number W05460602WC). One of 2 dried spotted bloods was tested by CLIP-PCR the next day in a molecular screen laboratory set up in the Nabang Township Hospital in 1st PL of YJC by the joint working group. All positives of CLIP-PCR or RDT were followed up with microscopy examination.

#### Vector surveillance

The data of + 1 area of Myanmar were collected from the monitoring records of the joint working group whom conducted periodic vector surveillance from June to September in 2018 and 2019. Ja Htu Kawng Village and 2 IDP camps were selected as capture sites, and then a house or a shelter was fixed as a capture point in the East, West, North, South and Middle of each capture site. Briefly, CDC light trap was hanged in indoor of each point; a 3-overnight capture was conducted in every 15 days from June to September and started before implementing indoor residual spraying (IRS) in June. The data of 1st PL of YJC were collected from county CDC, which carried out the national malaria vector surveillance project in 1st PL of YJC since 2015 with the same measures as + 1 area of Myanmar. The data of *Anopheles minimus* (*An. minimus)* females were extracted.

### Statistical analysis

The databases were generated with Microsoft Excel 2010 and analyzed with Statistical Product and Service Solutions (SPSS) software version 20. Descriptive statistics was used and the statistically significant differences (SSD) of incidence or rate were compared with chi-square test and odds ratio (OR) with 95% confidence interval (CI). The level of significance was set at *p* value ≤ 0.05 and (1-OR) multiply 100 were used to indicate the degree of reduction or increment. To assess the case detection, API (number of new parasitological confirmed malaria case per 1000 population per year [24]) with 95% CI was computed as a key indicator in + 1 area of Myanmar, whereas annual importation rate (AIR, number of registered to imported case per 1000 population per year) with 95% CI was computed after getting rid of indigenous cases in YJC of China and TPLs. Both SSD were analyzed by comparing with last year and between 2019 and 2016, respectively. Annual blood examination rate (ABER), malaria test positivity rate (MTPR) and proportion of *Plasmodium vivax* (*P. vivax*) were also evaluated. The parasite prevalence rate (PPR, number of positive cases per 100 investigated participants) with 95% CI was calculated by using PCR, microscopic and submicroscopic, SSD were compared between the submicroscopic PPR of the two surveys. The monthly density (number of females per trap per night per month) of *An. minimus* was calculated and its reduction rate was calculated by using the averaged density in June and September between 2018 and 2019. The correlation between monthly density and number of malaria cases was measured with bivariate Pearson correlation, and SSD of the density between + 1 area of Myanmar and 1st PL of YJC was compared with rank sum test of nonparametric tests.

## Results

### Case detection

**Fig. 2:**
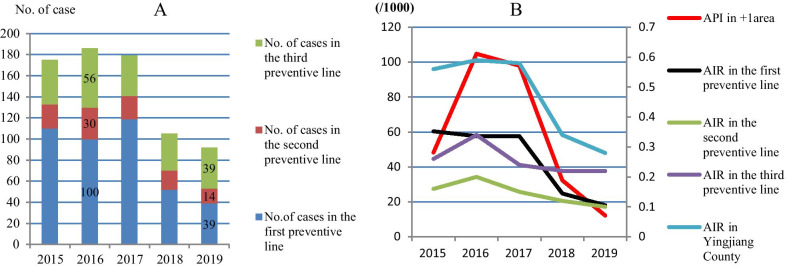
The trend of annual parasite incidence in + 1 area of Myanmar, and the annual importation rate and cases composition in Yingjiang Country of China from 2015 to 2019. **a** The composition of imported cases in the three preventive lines from 2015 to 2019. **b** The trend of API in + 1 area of Myanmar and AIR in the three preventive lines and Yingjiang County from 2015 to 2019. *Note* (1) *API* annual parasite incidence; (2) *AIR* annual importation rate

In + 1 area of Myanmar, a total of 5847 cases were confirmed during 2015 to 2019, in which *P. vivax* accounted for 96.27% (5629/5847) and *P. falciparum* 3.73% (218/5847). The averaged API was (59.11 ± 40.73)/ 1000, all of API had SSD compared with the previous year and between 2019 and 2016. The API dropped from 104.77/1000 in 2016 to 12.18/1000 in 2019 with a reduction of 89% (95% CI 88–91%, Table 1). In YJC of China, a total of 737 cases were registered from 2015 to 2019, in which 98.37% (725/737) was *P. vivax* and 1.63% (12/737) was *P. falciparum*; 0.95% (7/737) was indigenous cases and 99.05% (730/737) was imported cases. Among the seven indigenous cases, six were in 2015 (five in 1st PL and one in 2nd PL) and one was in 2nd PL in 2016. There have been neither new indigenous cases nor introduced cases since 2017. Among the 730 imported cases, only one case of 3rd PL came from Thailand in 2015, the rests imported from Myanmar. The proportion of reported cases by TPLs in the total cases of YJC was 56.99% of 1st PL, 14.52% of 2nd PL and 28.49% of 3rd PL. The averaged AIR was (0.47 ± 0.15)/1000, all had SSD compared between AIR in 2019 and 2016.The AIR dropped from 0.59/1000 in 2016 to 0.28/1000 in 2019 with a reduction of 53% (95% CI 39–63%) in YJC, meanwhile, it dropped from 57.63/1000 to 18.01/1000 with a reduction of 67% (95% CI 52–77%) in 1st PL, from 0.20/1000 to 0.10/1000 with a reduction of 52% (95% CI 10–75%) in 2nd PL and from 0.32/1000 to 0.22/1000 with a reduction of 36% (95% CI 3–57%) in 3rd PL, respectively (Table1). The trend of case detection in the study areas from 2015 to 2019 were depicted in Fig. 2.

**Table 1:** The results of case detection in each study area of the pilot project

Area	Year	Population	No. of blood examination	No. of confirmed or registered cases	Proportion of *Pv* (%, No.)	ABER (%)	MTPR (%)	API or AIR (‰, 95% CI)	Compared with last year		Compared between 2019 and 2016	
									*X*^2^ (*P* value)	OR (95% CI)	*x*^2^ (*P* Value)	OR (95% CI)
+ 1	2015	19,470	5361	940	99.36 (934)	27.53	17.53	48.28 (45.27–51.29)	–	–	1462.89 (< 0.001)	0.11 (0.09–0.12)
	2016	19,583	9275	2080	97.69 (2032)	47.36	22.43	104.77 (100.51–109.03)	442.43 (< 0.001)	2.31 (2.13–2.5)		
	2017	19,754	8754	1936	98.19 (1901)	44.32	22.12	98.01 (93.86–102.15)	4.97 (0.026)	0.93 (0.87–0.99)		
	2018	20,560	8095	664	95.63 (535)	39.37	8.20	32.3 (29.88–34.71)	720.97 (< 0.001)	0.31 (0.28–0.34)		
	2019	18,640	7644	227	100 (227)	41.01	2.97	12.18 (10.6–13.75)	178.13 (< 0.001)	0.37 (0.32–0.43)		
The first preventive line	2015	1822	1163	110	100 (110)	63.83	9.46	60.37 (49.43–71.32)	–	–	36.84 (< 0.001)	0.33 (0.23–0.48)
	2016	1897	1293	100	100 (100)	68.16	7.73	57.63 (46.92–68.34)	0.43 (0.512)	0.91 (0.69–1.21)		
	2017	2067	1369	119	96.64 (115)	66.23	8.69	57.57 (47.52–67.62)	0.45 (0.504)	1.1 (0.84–1.44)		
	2018	2097	1424	52	98.08 (51)	67.91	3.65	24.8 (18.14–31.46)	28.39 (< 0.001)	0.42 (0.3–0.58)		
	2019	2165	1415	39	100 (39)	65.36	2.76	18.01 (12.41–23.62)	2.35 (0.126)	0.72 (0.47–1.1)		
The second preventive line	2015	141,107	4989	23	100 (23)	3.54	0.46	0.16 (0.09–0.22)	–	–	5.38 (0.020)	0.48 (0.25–0.90)
	2016	144,339	5532	30	96.67 (29)	3.83	0.54	0.20 (0.13–0.27)	0.81 (0.368)	1.29 (0.74–2.24)		
	2017	147,084	5001	22	95.46 (21)	3.40	0.44	0.15 (0.09–0.21)	1.39 (0.239)	0.72 (0.42–1.25)		
	2018	148,477	5353	18	94.44 (17)	3.61	0.34	0.12 (0.07–0.18)	0.44 (0.508)	0.81 (0.43–1.51)		
	2019	145,741	4772	14	100 (14)	3.27	0.29	0.1 (0.05–0.15)	0.43 (0.513)	0.79 (0.39–1.59)		
The third preventive line	2015	158,872	12,553	42	97.62 (41)	7.90	0.33	0.26 (0.18–0.34)	–	–	4.52 (0.033)	0.64 (0.43–0.97)
	2016	165,853	13,785	56	96.43 (54)	8.31	0.41	0.34 (0.25–0.43)	1.44 (0.229)	1.28 (0.86–1.91)		
	2017	160,314	12,395	38	97.37 (37)	7.73	0.31	0.24 (0.16–0.31)	2.86 (0.091)	0.7 (0.46–1.06)		
	2018	161,782	10,005	35	100 (35)	6.18	0.35	0.22 (0.14–0.29)	0.15 (0.696)	0.91 (0.58–1.44)		
	2019	179,325	6553	39	100 (39)	3.65	0.60	0.22 (0.15–0.29)	0.001 (0.982)	1.01 (0.64–1.59)		
Yingjiang County	2015	301,801	18,705	175	99.43 (174)	6.20	0.94	0.56 (0.48–0.64)	–	–	35.82 (< 0.001)	0.47 (0.37–0.61)
	2016	312,089	20,610	186	98.39 (183)	6.60	0.90	0.59 (0.51–0.68)	0.29 (0.592)	1.06 (0.86–1.30)		
	2017	309,465	18,765	179	96.65 (173)	6.06	0.95	0.58 (0.49–0.66)	0.06 (0.815)	0.98 (0.79–1.2)		
	2018	312,356	16,782	105	98.1 (103)	5.37	0.63	0.34 (0.27–0.4)	19.99 (< 0.001)	0.58 (0.46–0.74)		
	2019	327,231	12,740	92	100 (92)	3.89	0.72	0.28 (0.22–0.34)	1.57 (0.210)	0.84 (0.63–1.11)		

(1) *Pv* *Plasmodium vivax*; (2) *ABER* annual blood examination rate; (3) *MTPR* malaria test positivity rate; (4) *API* annual parasite incidence; (5) *AIR* annual importation rate which was calculated after getting rid of the indigenous cases; *CI* Confidence interval; (6) *x*^*2*^ = chi-square test; (7) *OR* odds ratio

### Determination of parasite prevalence

In the first survey in April 2017, a total of 5570 participants including 37 febrile subjects were screened and 3 were RDT positives among the febrile ones. A total of 83 were found positive by CLIP-PCR, giving a PCR PPR of 1.49% (95% CI 1.17–1.81%, Table 2). Among the 83 PCR positives only 19 were positive by microscopy and all were *P. vivax*, giving a microscopic PPR of 0.34% (95% CI 0.19–0.49%), and 77.10% (64/83) of all infections were negative by microscopy with a submicroscopic PPR of 1.15% (95% CI 0.87–1.43%). The 3 RDT positives were both positive by PCR and microscopy. In the second survey in December 2019, a total of 1992 participants were screened and no one was RDT positive. 33 CLIP-PCR positives were identified with a PCR PPR of 1.66% (95% CI 1.1–2.22%). All 33 were microscopic negative, thus 100% of all infections were submicroscopic. The averaged PCR PPR was 1.58% in + 1 area. There was no SSD between the submicroscopic PPR of the two surveys and the microscopic PPR was reduced by 100%.

**Table 2:** The results of parasite prevalence survey in + 1 area of Myanmar

Date	No. of participants	CLIP-PCR		Microscopic		Submicroscopic		Submicroscopic PPR compared	
		No. of positives	PPR (%, 95% CI)	No. of positives	PPR (%, 95% CI)	No. of negatives	PPR (%, 95% CI)	*x*^2^ (*P* value)	OR (95% CI)
Apr.2017	5570	83	1.49 (1.17–1.81)	19	0.34 (0.19–0.49)	64	1.15 (0.87–1.43)	2.99 (0.084)	1.45 (0.95–2.21)
Dec.2019	1992	33	1.66 (1.1–2.22)	0	-	33	1.66 (1.1–2.22)		

(1) *PPR* parasite prevalence rate; (2) *CLIP-PCR* Capture and Ligation Probe-PCR; (3) *CI* confidence interval; (4) *OR* odds ratio

### Vector surveillance

**Fig. 3:**
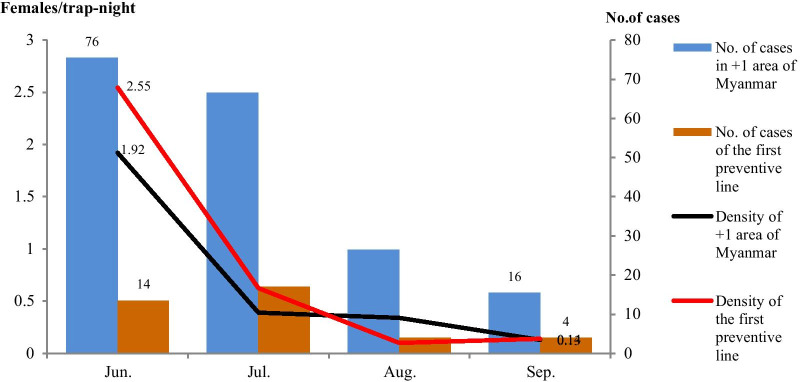
The trend of the averaged monthly density *of A. minimus* and No. of monthly malaria cases in June to September in 2018 and 2019. *Note* All house indoor residual spraying in the middle of June

A total of 440 female anopheles belonging to 10 species were captured in 2018 and 2019, which included *An. minimus*, *An. sinensi, An. jeyporiensis, An. maculatus*, *An. culicifacies*, *An. subpictus*, *An. barbirostris, An. barbumbrosus, An. messeae* and *An. bengalensis*. In which *An. minimus* and *An. sinensis* were malaria vectors and accounted for 66.14% (291/440) and 3.64% (16/440), respectively. *An. minimus* accounted for 63.70% (186/292) in + 1 area of Myanmar and 70.95% (105/148) in 1st PL of YJC. After pilot project, the averaged monthly density of *An. minimus* per trap-night dropped from 1.92 in June to 0.13 in September with a reduction of 93% in + 1 area of Myanmar, and from 2.55 in June to 0.14 in September with a reduction of 94% in 1st PL of YJC. The monthly density of *An. minimus* in + 1 area of Myanmar had a significant positive correlation with monthly number of malaria case (r = 0.74, *p* = 0.04), but no correlation was found in 1st PL of YJC (r = 0.45, *p* = 0.25). There was no SSD between the densities of both sides. The trend of averaged monthly density of *An. minimus* and monthly number of malaria cases was summarized in Fig. 3.

## Discussion

It is important to note that the National Malaria Control Programme in Myanmar was launched in 2016 and its goal is to eliminate malaria by 2030 [25, 26]. However, National Malaria Elimination Programme in China was initiated in 2010 and its elimination goal is by 2020 [1–3]. It is a 10-year difference involving distinct stages in achieving the goal of elimination malaria between China and Myanmar. Although the Myanmar national programme has included KSR II, the support from national programme barely covered KSR II in Myanmar due to the conflict with central government since 2012 [27]. This situation resulted in an imbalance of border malaria control capacities between China and Myanmar, and malaria from KSR II threatens the border areas of YNC.

In order to reduce malaria transmission and enhance the control capacities in KSR II, the pilot project on 3 + 1 strategy of joint cross-border malaria prevention and control was initiated in both sides and supported by local funds. Due to limited funding and human recourses, + 1 area of Myanmar and 1^st^ PL of YJC was set in a defined region, the width was limited to 2.5 km from boundary that was based on the fact that *An. Minimus* is dominant malaria vector in this area [28–30], with its maximum flight distance being 2.32 km [31, 32]. The purpose was to prevent mosquito transmission cross or along boundary by reducing vectorial capacity and building a flight buffer.

In 2016 in the + 1 area of Myanmar, the API unusually increased 2.31 times higher than 2015 (OR 95% CI 2.13–2.50, *p* value < 0.001), matching the WHO description on malaria outbreak (Fig. 2 and Table 1) [33]. Usually, malaria outbreaks in the Greater Mekong Sub-region are mostly ascribed to population movement and rarely to climatic factors [25]. In spite of the fact that difference of population between 2015 and 2016 was little (Table 1), and the rate of bednet ownership and proportion (97.3% in 2013) of sleeping under bednet was high [27, 34], especially sleeping under LLINs/ITNs (76.1% in 2013) [27], malaria outbreak unexpectedly occurred during the implementation of local GF malaria program in 2016. Several reasons could explain this, including that most of male adult conscribed into the ethnic army and stationed in the forest where malaria transmission risk was high [25], and when returning home they carried malaria back to communities. Another possibility was that usage of LLINs had expired [27]. When scaling up the proportion of LLINs distribution, coverage and use in the catchment areas of Myanmar was during the tenth round China GF malaria program from 2011 to 2013, and those LLINs expired by 2015 [27]. The IDPs moving into local GF malaria program did not have enough LLINs to replace them again and rarely implemented IRS and treated bednets due to funding gaps, leading to potential rebound of malaria transmission to pre-existing level [23].

Our findings showed that 96.27% of confirmed cases was *P. vivax* in the studied area, which is more difficult to control or eliminate than *P. falciparum* due to its several distinct biological characteristics [35], such as gametocytes in peripheral blood being matured to transmit to merozoites before symptoms appear; *P. vivax* malaria parasite density being more likely too low to be detected by microscopy or RDT; and hypnozoites in liver cell causing multiple relapse and requiring a 14-day-course treatment of primaquine which patients may not fully adhere. Our implemented pilot project had remarkable results in controlling malaria outbreak in + 1 area of Myanmar, most likely because of core WHO recommended malaria interventions and classical Chinese measures such as radical treatment at the resting stage [24, 35] (Table 1).

In the first year (2017) of the pilot project, vector control in + 1 area of Myanmar was one net per bed to distribute new LLINs provided by Health Poverty Action (HPA). IRS and vector surveillance were not carried out due to preparation of materials and documents, other interventions were performed since April 2017. Our findings showed the API in + 1 area of Myanmar in 2017 had SSD than 2016 (*p* value = 0.026). This meant the results of malaria control in + 1 area of Myanmar in the first year was effective, but the API only reduced by 7% (95% CI 1–13%), malaria transmission was still high. In the middle of June in the second (2018) and third (2019) years, all houses in + 1area of Myanmar and 1st PL of YJC were simultaneously treated with IRS using high-efficiency cypermethrin with WHO recommended dosage [36], and malaria vector was periodically monitored from the early of June to end of September, while the other interventions were maintained the same as 2017. The results of malaria control in + 1 area of Myanmar in the past two years had notable achievements, as API was sharply reduced by over 60% due the combined effect of IRS and LLINs.

The proportion of *An. minimus* in the female anopheles in + 1 area of Myanmar was as high as 63.7% and had a positive correlation with number of malaria case (r = 0.74, *p* = 0.04), which unequivocally confirmed again that *An. minimus* was the predominant malaria vector in this area. Previous study showed this mosquito rests indoor, prefers human blood and the biting peaks are in the sunset and midnight [37]. In our study, we chose the monthly density of *An. minimus* to evaluate the effect of IRS and LLINs and used the averaged monthly density of 2018 and 2019 to eliminate the climate impact. Generally, IRS protects residents against this mosquito biting before going to bed by killing mosquito that rest indoor, whereas LLINs protect residents after they go to bed by killing mosquito that rest on bednets. Both interventions had high use coverage in + 1 area of Myanmar and might have resulted in significantly reduced vectorial capacity and transmission. Figure 3 showed the averaged monthly density of *An. minimus* sharply reduced in July after implementing IRS, with corresponding sharp reduction of the number of malaria cases in August. IRS and LLINs/ITNs are core interventions of vector malaria control recommended by WHO [24], both may be less effective in reducing *P. vivax* transmission. But our findings revealed that they had a significant positive effect in reducing *An. minimus* vectorial capacity and competency.

Measuring the accurate parasite prevalence in the low transmission areas can be challenging due to limited sensitivity of microscopy and RDT [24]. Using CLIP-PCR, a high-throughput and highly sensitive molecular assay with a limit of detection of 0.01 parasites/μl [22, 23], we showed that the averaged PCR PPR was 1.58% in + 1 area of Myanmar. Additionally, our finding indicated the averaged API in + 1 area of Myanmar was (59.11 ± 40.73) /1000 and ratio of *P. falciparum*/*P. vivax* was 0.04, therefore the area belongs to a very low transmission area according to the WHO category [24]. Furthermore, 64 of 83 PCR positives (77.10%) were submicroscopic infections (all were subsequently identified as *P. vivax* by fluorescence quantitative PCR), a rate similar to the submicroscopic *P. vivax* infection rate found by Moreira et al. [38] and Cheng et al. [39]. Our findings showed the PPR of microscopy was reduced by 100%, which proved the core interventions of case detection were very effective and local GF malaria program worked in a high ABER (Table 1). There was no SSD between submicroscopic PPR at the beginning and the end of the pilot project, supporting the notion of submicroscopic infection being as infectious parasite reservoir [41]. These findings prompted contextual adoption of targeted interventions to control or eliminate this infection reservoir. Examples of such interventions include tracking those who had the same trip as the case and administering the same treatment, administering a dose of chemprophylactics for case neighbors and families, or taking radical treatment for *P. vivax* case at the resting stage. Our finding also hinted that the source of infection still existed in + 1 area of Myanmar, once the vector control was weakened, malaria transmission could rebound again.

Interestingly, our findings showed that the number of cases reported by 1st PL accounted for 57% of the total cases in YJC and its AIR determined the trend of AIR in YJC. Meanwhile, Fig. 2 and Table 1 showed that except in 2018, the AIR in 1st PL reduced significantly correlating with the decrease of API in + 1 area of Myanmar (*p* value < 0.001), suggesting that most of the imported cases in 1st PL of YJC were from + 1 area of Myanmar, whereas 2nd PL and 3rd PL might come from Myanmar outside + 1 area. An explanation could be the annual cross-border population of YJC was relatively stable in recent years, and only small groups of population at risk (PAR) stayed overnight or lived abroad. Usually, PAR was certain residents of 1st PL and 2nd PL who long-rent the land of KSR II close to the border for crop cultivation, while a few of non-local and 3rd PL went to the interior of KSR II for planting and mining. However, among the border of 214.6 km between YJC and KSR II, only LZC was the most serious malaria epidemic area and transmission was perennial with seasonal peak due to low altitude and dense population, the other areas of KSR II were low epidemic areas and transmission was interrupted in the cold season due to high altitude. In particular, most of the PAR in 1st PL lived in foothill and lowland plantations within + 1 area of Myanmar had high malaria transmission, which were relatively isolated and far away from Burmese villages [27].

Also the malaria consultation post in 1st PL of YJC was still working normally which established by GF malaria program since 2007. Most of PAR would take a dose of chemprophylactics before leaving the country and the new LLINs were freely available. That was why the AIR in 1st PL from 2015 to 2017 showed little difference and did not change with API of + 1area of Myanmar, but it sharply reduced in API after implementing all house IRS in 2018, then stable in 2019 due to a part of PAR lived outside + 1 area of Myanmar where no IRS. The research also found that all had SSD compared AIR in YJC and TPLs between 2019 and 2016 and proved the strategy of TPLs and its targeted local community’s measures for each preventive line were effective.

Our study limitations include the average ABER was 40% of + 1 area and 66% of 1st PL, they were much higher than 2nd PL and 3rd PL due to the number of malaria tested in + 1 area including the number of tested in passive case detection and active case detection combined in report monthly. However, that number in TPLs was only passive case detection, not included active case detect. So, there was no comparability between + 1 area of Myanmar and TPLs of YJC. Furthermore, passive case detection in 1st PL detected all febrile patients, but 2nd PL and 3rd PL detected only 3 types of fever such as malaria fever, suspected malaria fever and fever of unknown cause. Bias due to the number of cases reported by 3rd PL was more than 2nd PL, this was because the rich people and non-local mobile population prefers to go to county-level hospitals where they could get better medical resources, as part of cases were transferred from 1st PL and 2nd PL to the county hospital for treatment. Additionally, although the malaria data in + 1 area of Myanmar were collected from the office of GF malaria program via the health information system, the data was monthly reported by the primary level and no any the detail information of individual case, therefore, the population information of the case cannot be further analyzed.

## Conclusion

The 3 + 1 strategy has made remarkable achievements and a buffer zone of border malaria was successfully established between YJC of China and LZC of Myanmar. In + 1 area of Myanmar, the integrated malaria preventive and control measures achieved significant results with the API and microscopic PPR reduced by 89% and 100% respectively. In YJC of China, the focalized approaches of malaria elimination for each preventive line showed significant milestones in local transmission interruption since 2017 with a county-wide AIR reduction of 53%.

## Supplementary Information

**Additional file 1: Material 1.** The Data of map about the study area of pilot project. **Material 2.** Data of Fig. 2. **Material 3.** Data of Fig. 3

## Data Availability

The datasets used and/or analyzed during the current study are available from the corresponding author on reasonable request.

## Abbreviations

ABER: Annual Blood Examination Rate
AIR: Annual Importation Rate
API: Annual Parasite Incidence
CDC: Center for Disease Control and Prevention
CI: Confidence interval
CLIP: Capture and ligation probe
GF: Global Fund to Fight AIDS, Tuberculosis and Malaria
IDP: Internally displaced persons
ITNs: Insecticide treated nets
KSR II: Kachin Special Region II
LLINs: Long-lasting insecticidal nets
LZC: Laiza City
MTPR: Malaria Test Positivity Rate
OR: Odds ratio
PAR: Population at risk
PCR: Polymerase chain reaction
PL: Preventive line
PPR: Parasite Prevalence Rate
RDT: Rapid Diagnosis Test
SSD: Statistically significant differences
TPLs: Three preventive lines
WHO: World Health Organization
YJC: Yingjiang County
YNC: Yunnan Province of China
