# Supplementary material for: Effectiveness of joint 3 + 1 malaria strategy along China–Myanmar cross border areas
Source: BMC Infect Dis. 2021 Dec 14;21:1246. doi: 10.1186/s12879-021-06920-z (PMC8670156; doi:10.1186/s12879-021-06920-z)
Supplement: Supplementary file 1 — Additional file 1: Material 1. The Data of map about the study area of pilot project. Material 2. Data of Fig. 2. Material 3. Data of Fig. 3 [file 12879_2021_6920_MOESM1_ESM.docx]

| **Material 1 ：The Data of map about the study area of pilot project** | | | | | | | | |
| --- | --- | --- | --- | --- | --- | --- | --- | --- |
| **Region** | Range | **Components** | **Borderline（Km)** | **Latitude** | **Longitude** | **Altitude（m）** | **Population** | **Health Facilities** |
| +1 area | Zone of 20.5Km ×2.5Km along border in Laiza City of Kachin Special Region II,Myanmar | Laiza City | 20.5 | 24.76872457 | 97.56859837 | 290 | 6503 | Laiza Central Hospital，Laiza City Hospital，The Camp Clinic of Je Yang Hka, The Camp Clinic of Hpum Lum Yang |
|  |  | Mung Seng Yang Village |  | 24.72868600 | 97.55661100 | 177 | 748 |  |
|  |  | Ja Htu Kawng Village |  | 24.70784201 | 97.56714866 | 237 | 494 |  |
|  |  | Je Yang Hka IDP Camp |  | 24.69068739 | 97.56766467 | 270 | 8461 |  |
|  |  | Border School Tsang |  | 24.67423051 | 97.56697721 | 324 | 500 |  |
|  |  | Hpum Lum Yang IDP Camp |  | 24.66143100 | 97.57310900 | 342 | 3462 |  |
|  |  | Sut Mu Awng Village |  | 24.63707800 | 97.56625700 | 467 | 176 |  |
| The first preventive line | Zone of 20.5Km×2.5Km border area in Yingjiang County corresponding to "+1 "area of Myanmar | Jie Dao Village | 20.5 | 24.74921497 | 97.55847493 | 203 | 153 | Nabang Township Hospital, Malaria Consultation Post of Nabang Township，4 village clinics |
|  |  | Shang Tian Ba Village |  | 24.76252939 | 97.56901749 | 262 | 221 |  |
|  |  | Wang Jia Zhai Village |  | 24.73029635 | 97.56741697 | 240 | 176 |  |
|  |  | Jing Po Zhai Village |  | 24.72501600 | 97.56946100 | 230 | 321 |  |
|  |  | Ka Ya He Village |  | 24.72184400 | 97.57058400 | 211 | 126 |  |
|  |  | Qiao Tou Village |  | 24.71393614 | 97.57326715 | 209 | 119 |  |
|  |  | Dao Nong Village |  | 24.67034000 | 97.58588800 | 800 | 245 |  |
|  |  | Li Su Zhai Village |  | 24.72465500 | 97.56965300 | 207 | 102 |  |
|  |  | Nan Kai Shan Village |  | 24.65560256 | 97.58058750 | 762 | 254 |  |
|  |  | Lu Ding Shan Village |  | 24.64803388 | 97.58559647 | 850 | 380 |  |
| The second preventive line | The other border areas in Yingjiang County besides the first preventive line | Zhi Na Township | 191.2 | 25.15073684 | 98.12833319 | 1041 | 15937 | 8 township hospitals, 62 village clinics, 2 Malaria Consultation Posts |
|  |  | Nong Zhang Township |  | 24.61588448 | 97.88152051 | 816 | 50721 |  |
|  |  | Tai Ping Township |  | 24.66016085 | 97.85125756 | 812 | 31114 |  |
|  |  | Tong Bi Guan Township |  | 24.61380505 | 97.65594708 | 1341 | 6891 |  |
|  |  | Xi Ma Township |  | 24.75691044 | 97.70023645 | 1693 | 14237 |  |
|  |  | Ka Chang Township |  | 24.98629809 | 97.80093138 | 1224 | 9762 |  |
|  |  | Su Dian Township |  | 25.10684808 | 97.93789494 | 1700 | 8593 |  |
|  |  | Meng Nong Township |  | 24.90687103 | 97.90491992 | 1774 | 11222 |  |
| The third preventive line | The non-border areas in Yingjiang County | Zhan xi Township | 0 | 25.06668008 | 98.14995857 | 992 | 26038 | 6 township hospitals, 61 village clinics, Malaria Consultation Post of Tai Ping township. |
|  |  | Mang Zhang Township |  | 24.97238656 | 98.12356622 | 1003 | 13377 |  |
|  |  | Xing Cheng Township |  | 24.78045804 | 98.07643776 | 852 | 20112 |  |
|  |  | Jiu Cheng Township |  | 24.74445250 | 98.07575235 | 847 | 23420 |  |
|  |  | You Song Lin Township |  | 24.71039864 | 98.17886497 | 1910 | 15347 |  |
|  |  | Ping Yuan Township |  | 24.71010153 | 97.93181490 | 827 | 63488 |  |

Material2：Data of Fig2

| Year | No.of cases in the first preventive line | No. of cases in the second preventive line | No. of cases in the third preventive line | API in +1area of Myanmar | AIR in the first preventive line |
| --- | --- | --- | --- | --- | --- |
| 2015 | 110 | 23 | 42 | 48.28 | 60.37 |
| 2016 | 100 | 30 | 56 | 104.77 | 57.63 |
| 2017 | 119 | 22 | 38 | 98.01 | 57.57 |
| 2018 | 52 | 18 | 35 | 32.30 | 24.8 |
| 2019 | 39 | 14 | 39 | 12.18 | 18.01 |

| Year | AIR in the first preventive line | AIR in the second preventive line | AIR in the third preventive line | AIR in YJC | API in +1 area |
| --- | --- | --- | --- | --- | --- |
| 2015 | 60.37 | 0.16 | 0.26 | 0.56 | 48.28 |
| 2016 | 57.63 | 0.2 | 0.34 | 0.59 | 104.77 |
| 2017 | 57.57 | 0.15 | 0.24 | 0.58 | 98.01 |
| 2018 | 24.8 | 0.12 | 0.22 | 0.34 | 32.30 |
| 2019 | 18.01 | 0.1 | 0.22 | 0.28 | 12.18 |

Material 3：Data of Fig3

|  | Month | Density of +1 area of Myanmar | No. of cases in +1 area of Myanmar | Density of the first preventive line | No. of cases of the first preventive line |
| --- | --- | --- | --- | --- | --- |
| average | Jun. | 1.92 | 76 | 2.55 | 14 |
|  | Jul. | 0.39 | 67 | 0.63 | 17 |
|  | Aug. | 0.34 | 27 | 0.10 | 4 |
|  | Sep. | 0.13 | 16 | 0.14 | 4 |
